# Evolving concepts of the protein universe

**DOI:** 10.1016/j.isci.2025.112012

**Published:** 2025-02-13

**Authors:** Prakash Kulkarni, Lauren Porter, Tsui-Fen Chou, Shasha Chong, Fabrizio Chiti, Joseph W. Schafer, Atish Mohanty, Sravani Ramisetty, Jose N. Onuchic, Mick Tuite, Vladimir N. Uversky, Keith R. Weninger, Eugene V. Koonin, John Orban, Ravi Salgia

**Affiliations:** 1 Department of Medical Oncology, City of Hope Medical Center, Duarte, CA, USA; 2 Department of Systems Biology, City of Hope Medical Center, Duarte, CA, USA; 3 Division of Biology and Biological Engineering, California Institute of Technology, Pasadena, CA, USA; 4 National Center for Biotechnology Information, National Library of Medicine, National Institutes of Health, Bethesda, MD, USA; 5 Proteome Exploration Laboratory, Beckman Institute, California Institute of Technology, Pasadena, CA, USA; 6 Division of Chemistry and Chemical Engineering, California Institute of Technology, Pasadena, CA, USA; 7 Department of Experimental and Clinical Biomedical Sciences “Mario Serio”, University of Florence, Florence, Italy; 8 Center for Theoretical Biological Physics, Rice University, Houston, TX, USA; 9 Department of Physics and Astronomy, Rice University, Houston, TX, USA; 10 Kent Fungal Group, School of Biosciences, Division of Natural Sciences, University of Kent, CT2 7NJ Canterbury, UK; 11 Department of Molecular Medicine, Morsani College of Medicine, University of South Florida, Tampa, FL, USA; 12 Department of Physics, North Carolina State University, Raleigh, NC, USA; 13 W. M. Keck Laboratory for Structural Biology, University of Maryland Institute for Bioscience and Biotechnology Research, Rockville, MD, USA; 14 Department of Chemistry and Biochemistry, University of Maryland, College Park, MD, USA

**Keywords:** Biological sciences, Biochemistry, Protein, Structural biology

## Abstract

The protein universe is the collection of all proteins on earth from all organisms both extant and extinct. Classical studies on protein folding suggested that proteins exist as a unique three-dimensional conformation that is dictated by the genetic code and is critical for function. In this perspective, we discuss ideas and developments that emerged over the past three decades regarding the protein structure-function paradigm. It is now clear that ordered (active/functional) and disordered/denatured (and hence inactive/non-functional) represent a continuum of states rather than binary states. Some proteins can switch folds without sequence change. Others exist as conformational ensembles lacking defined structure yet play critical roles in many biological processes, including forming membrane-less organelles driven by liquid-liquid phase separation. Numerous diverse proteins harbor segments with the potential to form amyloid fibrils, many of which are functional, and some possess prion-like properties enabling conformation-based transfer of heritable information. Taken together, these developments reveal the remarkable complexity of the protein universe.

## Graphical abstract

**Figure:**
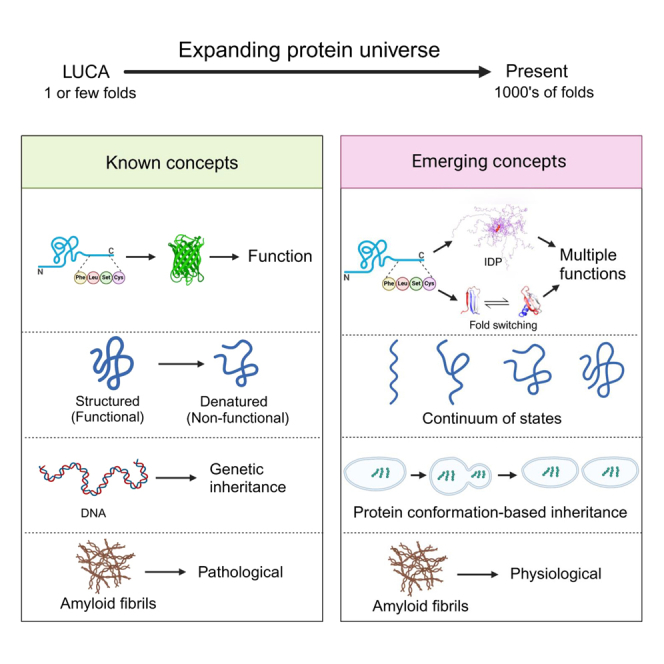

## Introduction

Proteins are responsible for the great majority of enzymatic, structural, transport, and signaling functions in every cell. Even the simplest bacterium or archaeon encodes hundreds of structurally and functionally distinct, diverse proteins, whereas complex multicellular organisms, plants, and animals typically encode tens of thousands of proteins, and this proteome is further expanded several folds through alternative splice forms and post-translational modifications. As such, proteins constitute a universe defined as the collection of all proteins from all organisms on earth, both extinct and extant.^1^ The protein universe is often described in terms of hierarchical models, although hierarchy is not necessarily its intrinsic property. Nonetheless, proteins are organized into tightly connected networks of homologous domains, suggesting that all proteins originated from a small number of ancestral folds.^2^^,^^3^ Deep evolutionary reconstructions indicate that the last universal cellular ancestor (LUCA) that is thought to have lived about 4 billion years ago already encoded at least several hundred proteins with diverse folds indicating that pivotal events in protein evolution occurred at very early stages of life’s history, antedating the emergence of “modern” cells.^4^

Proteins have been studied extensively across scales and disciplines to understand how amino acid sequence relates to a protein’s 3D structure and how that structure relates to its function. Observations dating back to the mid-1950s and later confirmed by X-ray crystallography, site-directed mutagenesis, and solution NMR, helped to firmly establish that most proteins have well-defined 3D structures. These observations led Anfinsen to postulate the “thermodynamic hypothesis”,^5^ which states that the protein’s native conformation is comprised of the totality of interatomic interactions which is determined by the amino acid sequence in a given environment.

However, classic work by Kauzmann (1959)^5^ explicitly refers to an alternative view of the protein folding problem: *“According to all that we know now about protein structure, we have good reason to believe that disorder might be introduced into a protein in small increments.”* In fact, by the 1970s, it was also recognized that proteins display complex behaviors and that protein structure can change in response to different aqueous environments.^6^^,^^7^ Nonetheless, for the purposes of protein structure analyses, it was generally held that proteins exist as binary states: ordered/folded (active) or denatured/unfolded (inactive).^8^ Subsequently, this concept was amended by the notion of intermediates between the folded and unfolded states. By the late 1980s and early 1990s, the energy landscape theory of protein folding and the funnel concept were developed.^9^^,^^10^ Per this theory, protein folding represents the progressive organization of partially folded structures in the conformational ensemble as that ensemble proceeds to the natively folded structure.^11^ As a result of evolution, proteins fold on rugged funnel-like landscapes biased toward the native structure.^12^^,^^13^ This funnel landscape theory captured how folding is related to function by showing not only how the precise structure can be attained but also how structural excitations can be involved in protein functionality.

The idea that excited states in the funnel landscape can have functional relevance became a major research topic, and in further major developments started in the 1990s, it became clear that proteins need not always be highly structured to be functional as presciently suspected by Kauzmann back in 1959.^5^ Indeed, it is now well recognized that a large fraction of the proteomes of organisms across all three domains of life is comprised of intrinsically disordered proteins/peptides (IDPs) and many more proteins with ordered domains contain intrinsically disordered regions (IDRs) that, by definition, lack rigid 3D structure yet are functional.^14^ Furthermore, it is now clear that some ordered proteins can switch folds and gain new function^15^ and that regions in certain folded proteins (and even entire proteins) can “unfold”, in a transition from order to disorder, in response to physical or chemical stimuli.^16^

**Figure 1:**
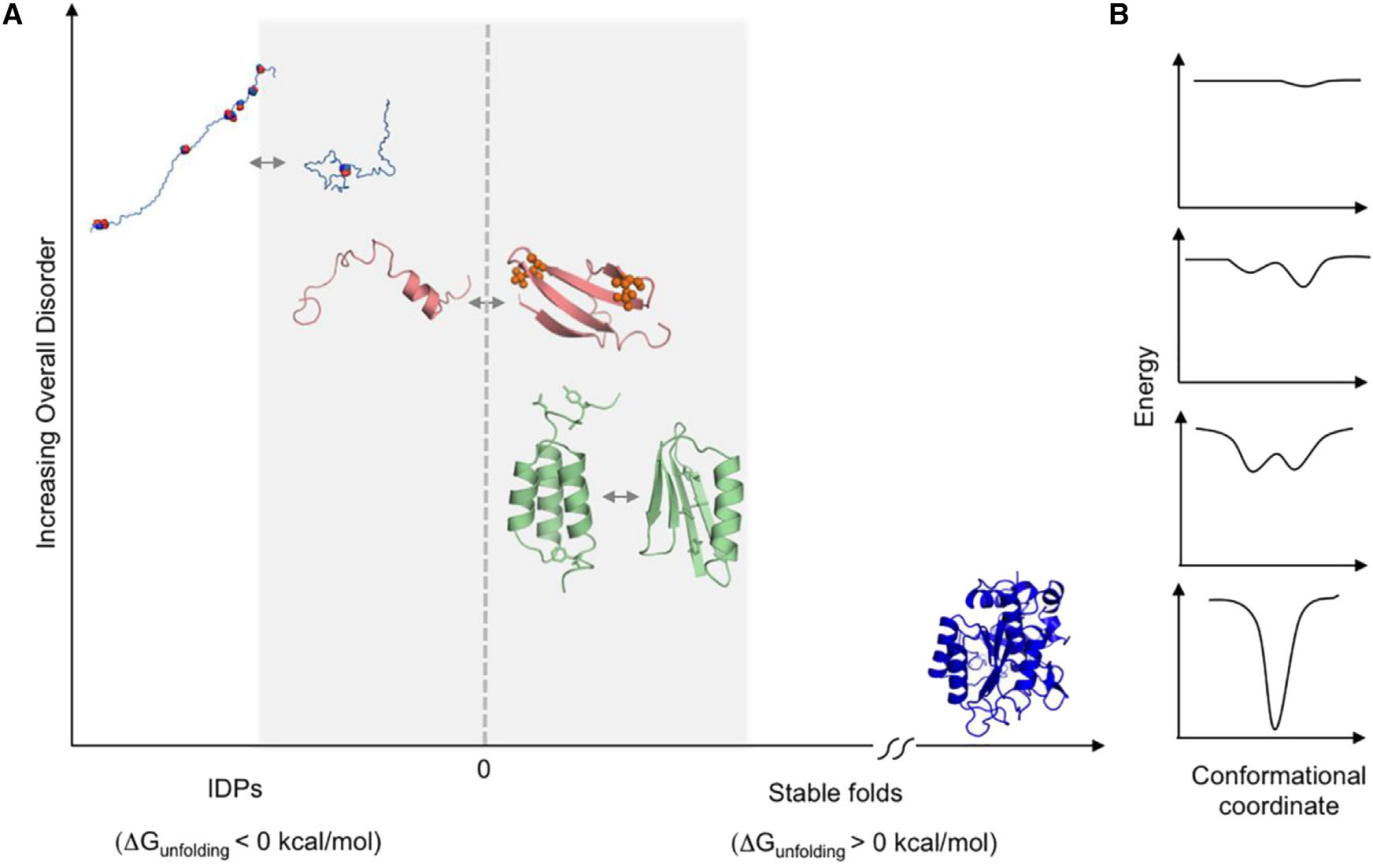
Proteins on the brink of stability can undergo a continuum of order/disorder transitions (A) Examples of transitions from top left to bottom right: transition between the extended and collapsed disordered states of prostate associated gene 4 (PAGE4), modulated by phosphorylation; 111 disorder-to-order transition of 4E-BP2 induced by phosphorylation; 23 order-to-order fold switching between GA98 and GB98, triggered by single amino acid changes or ligand binding.^21^ In contrast, stable proteins such as subtilisin (shown in dark blue) do not undergo such changes. (B) Approximate energy well diagrams for each protein from PAGE4 (top) to subtilisin (bottom) (from the study by Kulkarni P. et al.^20^ with permission).

Studies on fold switching in natural and experimentally designed systems showed that transitions occur when states have diminished stability.^17^^,^^18^-Fold switching involves flexible regions in one conformer or the other, and a new binding surface that when exposed in the alternate fold, can result both in stabilization of the alternative state as well as a change of the biological function. Detailed studies of IDPs/IDRs showed that, although IDPs can transition from disordered conformational ensembles to folded structures either prior to or upon binding to their cognate targets, many IDPs remain largely disordered even as they interact with the targets, and yet others exhibit dynamic excursions and stochastically switch conformational states while remaining disordered.^19^ Thus, IDPs may be only marginally unstable although they can be tipped to populate certain preferred conformations. The latter may populate a wide spectrum from a slight change in the ensemble conformation to a fully folded structure to gain functionality. Such changes in the structural ensemble of IDPs are conceptually similar to the fold switching events observed in marginally stable folded proteins in response to mutations or environmental triggers leading to new functions (Figure 1).^20^

Additional discoveries in the late 1990s further changed the canonical perception of protein structure/function when it became evident that, at least *in vitro*, most proteins or individual domains within them can convert into fibrils with all the characteristics of amyloid that accumulates in a variety of human and animal disorders.^22^ Consistent with this remarkable observation, it has been shown that fully soluble peptides and proteins, which have specific functions in this soluble state, can form amyloid fibrils representing distinct, well-defined functional states that are not necessarily pathological.^23^

The discovery of prions, a sub-class of amyloids in which protein aggregation becomes self-perpetuating and, at least, in well-studied cases, infectious, further challenged prevailing wisdom by demonstrating the existence of protein-based inheritance.^24^^,^^25^^,^^26^ Although originally met with disbelief, this form of inheritance has been found in diverse organisms in all three kingdoms of life, is now accepted as a *bona fide* epigenetic mechanism.^24^ Moreover, a groundbreaking study revealed that protein-based inheritance may not be particularly rare or restricted to amyloid-based prions.^26^ This study identified a new class of prion-like proteins that are capable of causing heritable, protein-based “molecular memories” that are sustainable over hundreds of generations yet are distinct from the classical amyloid prions.^26^

Although previously observed with certain ordered proteins, a more recent notable discovery demonstrated how IDPs can undergo liquid-liquid phase separation (LLPS) via multivalent protein-protein interactions, thereby driving the biogenesis of numerous membrane-less organelles (MLOs).^27^ Indeed, bioinformatics predictions suggest that the vast majority of IDPs harbor the propensity for LLPS. In this perspective, we discuss how protein structure and folding are fundamental to the inherent ability of proteins to switch between conformations, revealing essential but previously underappreciated features of the protein universe.

## Intrinsically disordered yet functional proteins

Two characteristic features of many IDPs/IDRs are low mean hydropathy and relatively high net charge, which are critical prerequisites for the lack of rigid 3D structure in proteins under physiological conditions. Some IDPs can transition to order under conditions such as low pH or heating that result in the denaturation or unfolding of ordered proteins while other IDPs lose function when folded, and the activation of yet other IDPs involves unmasking cryptic disorder.^28^ These properties suggest that the order/disorder equilibrium in IDPs is subtle and that, many IDPs are very close to this boundary. It has been proposed that IDPs are “edge of chaos” systems that operate in a state between order and randomness (chaos), where complexity is at its maximum.^19^ Furthermore, consistent with the edge-of-chaos concept, analysis of protein structures solved in different conditions and functional states revealed that hundreds of protein fragments, dubbed “dual personality fragments”, exist both as disordered and ordered states. These fragments display singular features that differentiate them from both folded proteins and IDPs/IDRs and are frequent targets of regulation, either by allostery or by post-translational modifications.^29^

It has also been demonstrated that IDPs can interact with picomolar affinity with partner proteins even while being fully disordered and highly dynamic.^30^ Furthermore, some IDPs/IDRs can fold differently upon interaction with different partners, thereby gaining distinct structures in a context-dependent manner.^31^^,^^32^ Many IDPs/IDRs can also form “fuzzy complexes” possessing high levels of disorder in bound states.^33^^,^^34^^,^^35^

As a result of all these discoveries, our view of protein interactions has come a long way from the concept of rigid structures. It is now understood that proteins form conformational ensembles of proteins which manifest in multiple modes of interactions between partner proteins. This context-dependence, referred to as multiplicity of binding modes, is achieved by sampling multiple minima of the interaction energy landscape.^36^ This situation has been observed in many protein systems where multiple conformations are necessary. Initially, multiplicity of binding modes was discussed in the context of dual funnel basins for functional reasons,^37^^,^^38^^,^^39^ but in many cases, multiple stable basins may exist.^40^ The possibility of multiple structures for the same protein is particularly notable in the case of IDPs.

Physiologically, IDPs participate in a plethora of biological functions where multiple partner interactions and high-specificity/low-affinity interactions are crucial. Thus, IDPs are involved in several key processes, such as transcription, translation, and signaling. IDPs are also critical to several higher-order phenomena, such as regulation of the cell division cycle, circadian rhythms, and phenotypic plasticity (see recent reviews).^14^^,^^41^^,^^42^ Here, we focus on those aspects that have contributed to the conceptual advancement of the IDP field in the past few years, namely IDP conformational noise and LPS and discuss their implications. We also discuss fold-switching proteins, which are conformationally heterogeneous like IDPs, but differ in stability, selection, and biological scope.^17^

Noise, that is, variation in cell-to-cell characteristics that occurs in populations of isogenic cells, at the levels of transcription, translation, post-translational modification, protein folding, and various signaling pathways, is now a well-recognized concept in biology.^43^ However, noise caused by IDP conformational dynamics has remained under-appreciated. A living cell is a complex adaptive system, a dissipative microenvironment that operates far from thermodynamic equilibrium. Operationally, a living cell is akin to a computer. Its hardware and software constitute the “wetware” attuned to logic gates allowing the cell to “make decisions”.^19^ In this metaphor, IDPs represent critical components of the wetware. They are “wired” to form the cell’s protein interaction network that is scale-free and minimally frustrated.^44^ The design and functionality of this network to a large extent are governed by the IDPs whose expression is tightly regulated. Because IDPs exist as ensembles of states populating an energy landscape, where the individual states are separated by relatively low energy barriers, or multiple conformations that are further influenced by specific post-translational modifications such as phosphorylation, IDP conformational dynamics manifests itself as conformational noise.^45^ This noise can potentially amplify transcriptional and translational noise to stochastically switch cellular phenotypes. Dysregulation of IDPs in response to intrinsic or extrinsic perturbations results in “rewiring” of the network and influences cell-fate decisions.^19^^,^^45^ Furthermore, such dysregulation of IDPs can trigger pathological states including cancer and neurodegenerative diseases.^46^ Given that ∼90% of transcription factors harbor IDRs, conformational noise is likely to comprise an integral part of transcriptional noise, underscoring its fundamental importance.^45^^,^^47^

## Disorder, LLPS, and MLOs

Probably the most graphic illustration of the emergent behavior based on protein intrinsic disorder is the LLPS phenomenon that has attracted considerable attention in recent years.^48^ Although LLPS *per se* has been known since the 19^th^ century, and it was also well known that folded proteins can undergo LLPS, the phenomenon has found new applications in the realm of biological functions of the IDPs. LLPS is thought to be the molecular mechanism underlying the emergence of numerous biomolecular condensates or MLOs, which represent distinct compartments in the cell cytoplasm or nucleoplasm that are not bounded by membranes, have a composition different from the surrounding cellular environment, and play a crucial role in spatiotemporal organization of the intracellular space. Nucleoli, stress granules, P bodies, pericentriolar material, paraspeckles, and germline P granules are all typical MLOs.^49^^,^^50^

MLOs obviate the necessity to facilitate and regulate molecular interactions via reversible and controllable sequestration of interacting molecules in specialized compartments.^51^ MLOs are open dynamic systems; they represent highly pliable complex networks that exist at the edge of chaos. They are characterized by unique structural and compositional dynamics. Furthermore, their multicomponent nature and polyfunctionality facilitate the fine regulation of many biological processes. MLOs are exquisitely sensitive to changes in external conditions. Even minor changes can result in constituent proteins aggregating irreversibly, transitioning to a gel-like state, or forming amyloid fibrils.^52^ The structural dynamics and fluidity of MLOs, their liquid-like characteristics, as well as their multifunctionality and responsiveness to various intra- and extracellular signals all originate from the specific IDPs/IDRs that serve as LLPS drivers.^53^ In line with these considerations, MLOs are enriched in IDPs and proteins containing IDRs,^51^ and their biogenesis was shown to be controlled by IDP concentrations, post-translational modifications, binding partners, and environmental perturbations.

Besides being important for cellular organization, MLOs or biomolecular condensates have been reported to play a role in many cellular processes, including transcriptional regulation, chromatin organization, and DNA damage repair.^27^^,^^54^ At the molecular level, the formation of functionally relevant condensates is often driven by dynamic, multivalent, selective interactions between IDRs.^27^^,^^55^^,^^56^ An increasing number of reports have documented that disease-associated mutations within IDPs can lead to dysregulation of their multivalent interactions and condensate formation, thereby contributing to the pathology of many diseases, including cancers and neurodegenerative disorders.^46^ Many IDPs that carry disease-associated mutations are involved in transcriptional regulation, e.g., fusion of oncogenic transcription regulators and polyQ expansion proteins associated with neurodegenerative diseases.^57^ Targeting these pathological condensates suggests potentially new and exciting therapeutic strategies.

## Same sequence but alternate folds: Fold-switching proteins

In 2008, the protein XCL1 was found to independently, and reversibly, interconvert between two conformations.^58^ In the 15 years since this initial discovery, the list of fold-switching “exceptions” to Anfinsen’s postulate has continued to grow. Now, fold switchers are a recognized class of proteins with unique biophysical characteristics and are estimated to comprise as much as ∼4% of the Protein Data Bank.^15^ Examples of other notable naturally occurring fold switchers include: (1) KaiB, an integral component of the cyanobacterial circadian clock,^59^ (2) RfaH, a member of the universally conserved NusG family of transcription factors,^60^ (3) Lysenin, a pore-forming toxin that switches folds upon membrane binding,^61^ and (4) ORF9b, a virulent SARS-CoV-2 protein that was found to fold-switch upon binding to human TOM70.^62^ To date, nearly 100-fold switchers have been experimentally characterized, several of which are implicated in diseases such as cancer, viral infection, bacterial infection, and autoimmune disorders.^63^

The increasing number of identified fold-switching proteins and their health implications have broadened researchers’ interests in the fundamental biophysical principles governing this phenomenon. Although fold switching is not prohibited by physical or chemical laws, a protein must assume a biologically active fold, and stable alternatives would act as kinetic traps.^64^ Such traps are expected to be avoided by fold-switching proteins via transformations that do not pass through fully unfolded states. Zuber et al.^21^ analyzed the mechanism of the fold-switching transformation in RfaH. RfaH is among the most dramatic fold-switchers, with 50 residues of its C-terminal domain reversibly interconverting between an α-hairpin and a β-barrel. This transformation occurs as a result of binding to opsDNA, and the refolding path of RfaH does not sample a fully unfolded state, reinforcing predictions made on how fold-switching mechanisms would avoid kinetic traps.^21^

**Figure 2:**
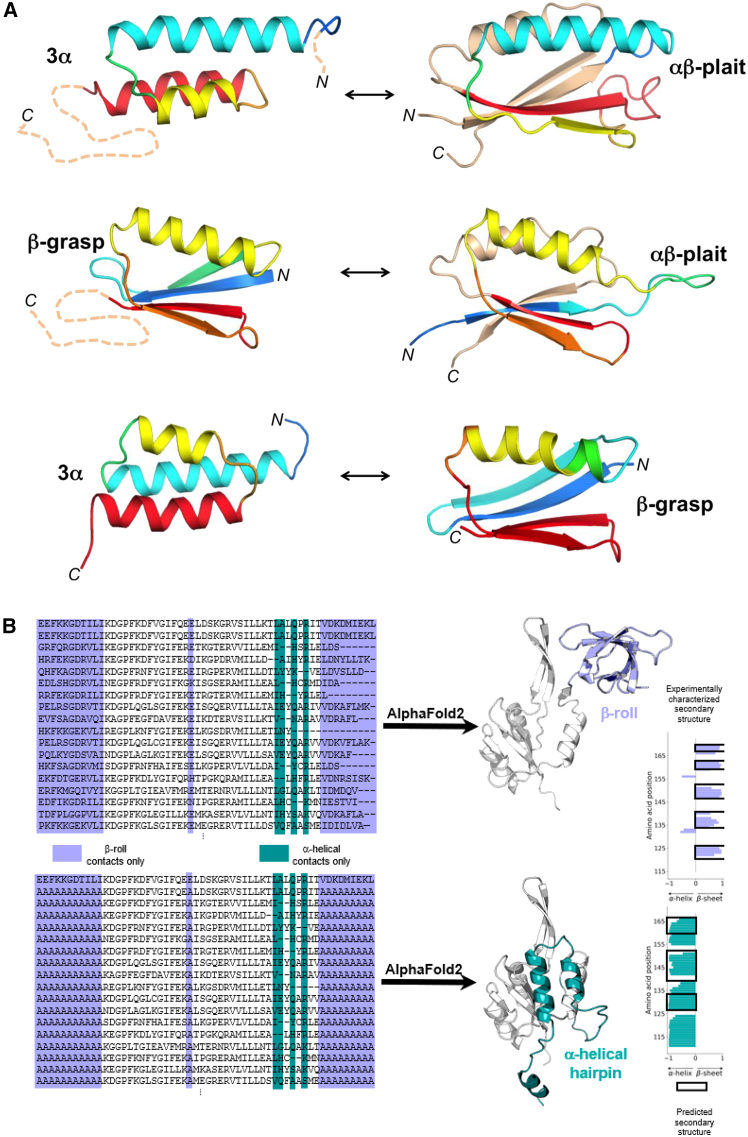
Fold-switching proteins (A) Summary of designed switches between 3α, αβ-plait, and β-grasp fold topologies that have been characterized structurally. Color-coding indicates how the corresponding regions change in the alternative fold. The dashed lines show regions that are disordered in one of the states but not the other. (B) Top, a NusG N-terminal (NGN) fold (light gray) and a C-terminal β-roll fold (lavender) are predicted from a deep input MSA (region corresponding to the CTD shown) generated from the sequence of a NusG protein with ≤29% aligned identity to its homologs with experimentally determined structures. Predicted β sheets in the C-terminal domain that agree closely with the β sheets predicted from nuclear magnetic resonance experiments are shown with black boxes surrounding lavender bars. Bottom, a NusG N-terminal (NGN) fold (light gray) and a C-terminal α helical hairpin fold (teal) are predicted from a modified input MSA of the NusG homolog in which columns predicted to form only β-roll contacts are changed to alanine. Predicted α helices in the C-terminal domain that agree with the α helices predicted from nuclear magnetic resonance experiments are shown with black boxes surrounding teal bars. Protein structures were generated with PyMOL43.

Fold switching is also being exploited to better understand how amino acid sequences encode both protein structure and function. Ruan et al.^65^ engineered mutational pathways between three common folds: S6 (a component of the 30S ribosomal subunit), GA, and GB, corresponding to α/β-plait, homeodomain-like 3α, and ubiquitin-like β-grasp folds, respectively (Figure 2A). Critical points in sequence space were identified where a single mutation changes the conformation and function of a protein. The existence of these critical points demonstrates that unique conformations are not necessarily separated by high barriers in the folding landscape.^66^ Furthermore, Solomon et al. engineered a single sequence that transitions between the GA (3α) fold and the S6 (α/β-plait) fold in response to temperature changes within a relatively narrow range.^18^ These protein engineering studies demonstrate the importance of disordered regions in remodeling ordered states. They also imply a naturally occurring mechanism of fold-switching evolution. Recent work has uncovered a stepwise mutational path that switched the helix-turn-helix fold of bacterial response regulators to a winged helix,^67^ demonstrating that natural proteins have also switched folds by stepwise mutation over evolutionary history.

Because fold-switching proteins challenge the single-fold paradigm that has shaped much of protein structure prediction, considerable interest has focused on predicting both conformations of fold-switching proteins from sequence. Machine learning algorithms, such as AlphaFold2, have revolutionized predictions of protein structure but miss nearly all (85/93) alternate conformations of fold-switching proteins using default settings and shallow random sequence sampling.^68^ Given that structure prediction by AlphaFold2 is based on evolutionarily conserved patterns in protein sequences, this discrepancy might be explained by fold switching being an infrequent evolutionary quirk.^69^ Alternatively, machine learning algorithms might miss coevolutionary signatures of alternative protein folds. To investigate this, Schafer and Porter developed alternative contact enhancement (ACE) to apply coevolutionary algorithms to known fold-switching proteins.^70^ They calculated coevolutionary couplings between amino acid pairs from multiple sequence alignments (MSAs) that represent protein superfamilies (deep MSAs that contain diverse-yet-homologous sequences) and subfamilies (shallower MSAs that preferentially retain sequences highly similar to the target protein). Applying this method to all known fold-switching proteins for which deep MSAs could be generated, they found unique coevolutionary signals for both conformations in all cases. These results suggest that fold switching confers an advantage that can be selected for during evolution, and that prediction of fold-switching proteins should be possible using strategies that rely on structural information derived from coevolution when enough sequence information is available.

Dual-fold coevolution paves the way for machine learning algorithms to predict 2-folds from one sequence more consistently. Accordingly, *in silico* alanine mutations introduced into the MSAs of dynamic single-fold proteins led to successful predictions of structural ensembles in several cases.^71^ This latter approach was tested on a NusG variant with 29% sequence identity to its closest homolog with known structure from Protein Data Bank. AlphaFold2 successfully predicts both its α-hairpin and the β-barrel conformations. These predictions are consistent with amino-acid-specific secondary structure calculated from NMR assignments^70^ (Figure 2B). There has also been success using a class of algorithms broadly called diffusion models to predict/engineer proteins.^72^ To explore diffusion models’ ability to sample the conformational ensemble, Jing et al. developed EIGENFOLD.^73^ Although its accuracy is competitive for single-fold predictions, it is not yet able to accurately capture both conformations of fold-switching proteins. Nevertheless, with these and other notable computational developments,^74^ it is expected that methods will be developed in the near future that will predict two 3D structures from one amino acid sequence consistently and accurately.

## Conversion of native proteins into amyloid fibrils

**Figure 3:**
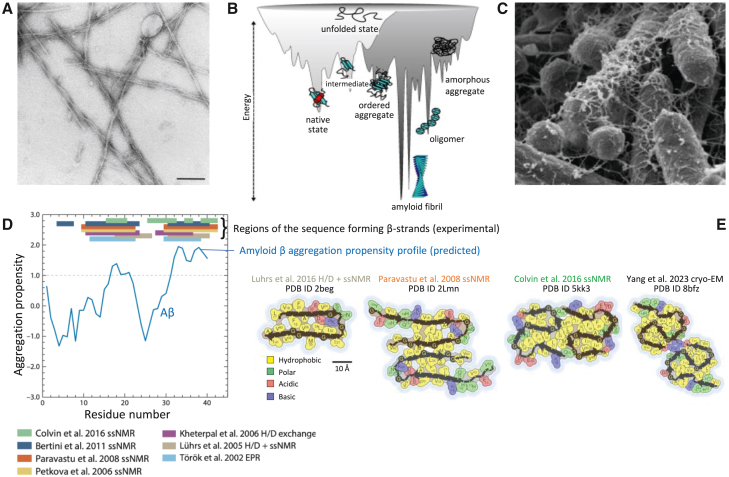
Amyloid-forming proteins (A) Amyloid-like fibrils formed from an SH3 domain. Readapted with permission from the study by Watson J. L. et al.^72^ (B) Energy funnel illustrating the protein folding (left) and the protein aggregation (right) components. Readapted with permission from the study by Parui S. et al.^74^ (C) Curli of bacteria visible as filamentous structures as an example of functional amyloids. Readapted with permission from the study by Olsen A. et al.^77^ (D) Comparison between predicted protein aggregation profile (blue) and regions of the sequence found experimentally to form β-strands in amyloid fibrils (colored bars). Readapted with permission from the study by Westermark P. et al.^75^ (E) The four structures on the right are cross-sectional structures of amyloid fibrils along with their PDB entries and references, as extracted from the amyloid atlas. Readapted with permission from the study by Fowler D.M. et al.^78^

The ability of certain proteins to convert from a soluble state—either folded or intrinsically disordered—into well-defined aggregates known as amyloid fibrils is now well documented. Amyloid fibrils are characterized by a fibrillar morphology, a β sheet, with parallel, in register β strands perpendicular to the fibril axis, and the ability to bind specific dyes such as Congo red and thioflavin T/S, have been known for over a century. Until 1998, however, it was thought that such fibrils were formed by only about 20 proteins which were known to be the main constituents of the amyloid deposits associated with well-defined pathological states, such as Alzheimer’s disease (AD), light chain amyloidosis (AL), familial amyloid polyneuropathy (FAP or ATTRv-PN), and several others.^75^ In 1998, however, the SH3 domain from the p85α subunit of bovine phosphatidylinositol 3-kinase was serendipitously found by Dobson and collaborators to form amyloids^76^ (Figure 3A). These amyloid fibrils were morphologically, structurally, and tinctorially indistinguishable from those associated with disease. When this discovery was reported, many in the field believed it was a peculiar behavior of the SH3 domain associated with its native all-β fold. However, the following year, a full-length α+β protein, human muscle acylphosphatase (mAcP), was found to undergo a similar conversion.^22^ These findings led to the novel concept that many, if not all, proteins can be induced to form amyloids *in vitro* under appropriate conditions, which found many experimental confirmations in the following 25 years, with over 100 proteins converted into *bona fide* amyloid-like fibrils including all-α proteins.

Amyloid formation is a distinct type of conformational switch of a protein from its native state (either folded or unfolded) to the amyloid fold dominated by a β sheet. Any organism must actively combat protein aggregation that would ultimately result in amyloid fibril formation because protein assemblies, particularly the protein oligomers formed early in the amyloidogenesis process, can be toxic. The protein homeostasis network, composed (at least) of the translational machinery, molecular chaperones, the ubiquitin-proteasome system (UPS) and the autophagy-lysosomal pathway (ALP), is now thought to prevent this deleterious, potentially pathological switch.^79^ The amyloid state is thermodynamically the most stable conformational state of a protein, and it is exactly where a protein ends up going if it is not attended by external factors that kinetically impede aggregation^80^^,^^81^ (Figure 3B). Moreover, there is not a unique amyloid fold that a specific protein can adopt, but multiple ones, known as polymorphs, which exacerbates the problem of thermodynamic stability and makes amyloid formation more favorable entropically^80^^,^^81^ (Figure 3B).

However, in addition to representing a pathological entity, amyloid fibrils can also constitute an ensemble of alternative functional states for proteins. The first evidence of a functional amyloid was obtained in 2002, only four years after the amyloid fold was discovered as a common structural state of proteins.^82^ In that work, the *Escherichia coli* CsgA curlin subunit was found to assemble *in vivo* and *in vitro* into fibrous structures, known as curli, with amyloid properties. Curli are anything but deleterious for bacteria; indeed, they have well defined, specific functions that allow bacteria to colonize inert surfaces and mediate binding to host proteins^82^ (Figure 3C). Other amyloid-associated functions have also been discovered in eubacteria, archaea, protists, fungi, plants, arthropods, and animals that is, across the tree of life.^83^ The first case of a human protein was found in 2006, when the intralumenal domain of Pmel17, a membrane protein of the melanosomes present in melanocytes, was shown to form fibrous striations in the form of amyloid fibrils on which the melanin polymers responsible for protecting human skin from damaging UV radiation are able to grow.^78^ Subsequently, a number of hormone peptides were found to accumulate as amyloids in exocytic vesicles, the pituitary secretory granules, before they are released as soluble peptides into the extracellular space.^23^ In this case, amyloid has a specific storage function, allowing the compaction of hormones before they are released in a controlled manner. The receptor-interacting serine/threonine-protein kinase 1/3 (RIP1/RIP3) also forms amyloid fibrils that mediate programmed cell necrosis induced by the tumor necrosis factor (TNF), extending the range of amyloid functions beyond the boundaries of structural roles to include signaling.^84^ Other cases then followed, making it clear that the amyloid switch represents an important functional resource in nature to generate new biological functions.

From a mechanistic standpoint, it is now clear that amyloid fibril formation occurs through a nucleated process where oligomers or even monomers that possess an appropriate conformation act as nuclei (primary nucleation) and recruit new monomers/oligomers to grow into a fibril with β sheet (elongation). Fibrils can also break into shorter yet growing fragments or catalyze the formation on their surface of new oligomers acting as nuclei (secondary nucleation), thereby augmenting their potential to propagate and diffuse.^85^ It soon became clear that specific amino acid segments of a peptide/protein sequence with a high β sheet propensity, high hydrophobicity, and low net charge promoted amyloid fibril formation and formed the β strands stacking along the fibril axis as shown here for Aβ (Figure 3D). In addition, mutations within these segments had major effects on the rate of amyloid formation.^83^^,^^86^ With the advent of dedicated applications of solid-state nuclear magnetic resonance (ssNMR) spectroscopy, and particularly cryo-electron microscopy (cryo-EM), many amyloid folds have been resolved to a high resolution which was unimaginable a few years ago. An amyloid atlas has recently been created,^87^ listing all such structures, along with representative images and references (https://people.mbi.ucla.edu/sawaya/amyloidatlas/). Polymorphism is a major feature of amyloids as the same peptide/protein can form several different amyloid structures (Figure 3E). Nevertheless, all of the structures present a β sheet structure, with parallel, in-register, β strands perpendicular to the fibril axis.^87^ Moreover, from a statistical survey of these structures, it is clear that amyloid-forming segments consistently have a high β sheet propensity, high hydrophobicity, and low net charge.

## Prions: Proteins as mediators of transgenerational inheritance

Within the ever-expanding family of amyloid-forming proteins, a remarkable subclass has emerged: prion-forming proteins, which add a paradigm-breaking dimension to the protein universe. When these diverse proteins refold into their amyloid form, they can mediate the flow of heritable information, which can be vertically transmitted across many generations Prions thus define a previously unsuspected, unique form of protein-only epigenetic inheritance. Furthermore, at least one prion is linked to several potentially fatal neurodegenerative diseases in humans and other mammals.

The first prion-forming protein to be described was mammalian PrPc. Its transmissible conformer (designated PrPSc) has been linked to several slow-manifesting, transmissible, fatal neurodegenerative diseases, typified by Creutzfeldt Jakob disease (CJD) in humans, bovine spongiform encephalopathy (BSE) in cows, and Scrapie in sheep.^88^ The original hypothesis proposed by Prusiner to explain the emergence and propagation of such a protein-only infectious agent postulated a form of self-templated structural conversion giving rise to highly stable and transmissible polymeric assemblies of PrPSc.^88^ The prion assemblies of PrPSc then act effectively as “proteopathic agents” that can spread within the infected host and can also be transferred between individuals of the same species. Prions are thus a unique class of infectious agents that challenges the accepted nucleic acid-based paradigm of heredity and infection.

**Figure 4:**
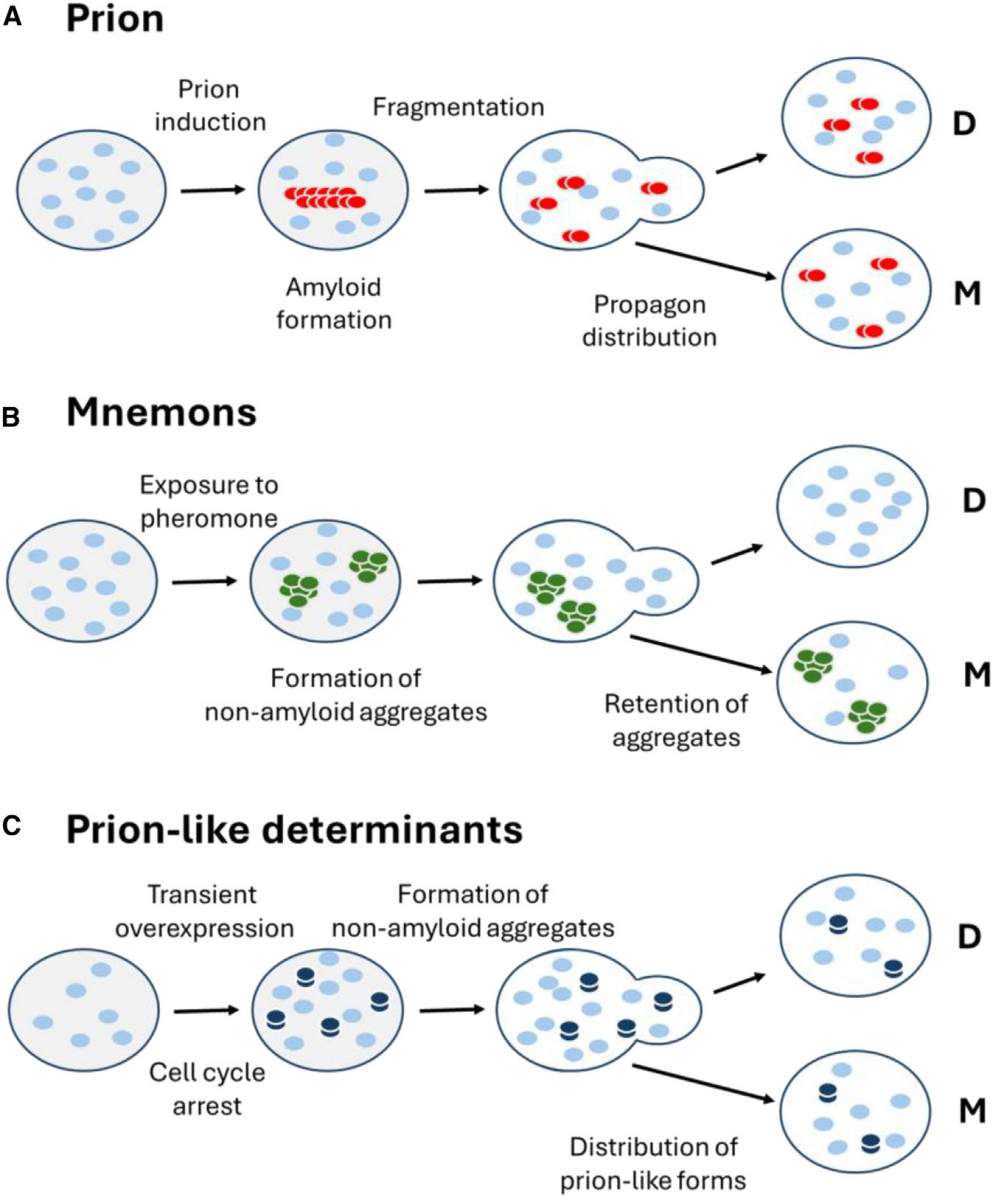
Three types of protein-based inheritance described in the yeast *Saccharomyces cerevisiae* (A) Prions that can be generated *de novo* by overexpression of the prion-forming protein. The resulting amyloid fibrils (red) are fragmented by Hsp104 into smaller elements called propagons that can be transferred to daughter cells at mitosis. (B) Mnemons are generated in cells in response to a chemical signal. The only example described in detail so far is the Whi3-based mnemons (ref.^90^), high-molecular-weight forms of the protein (in dark blue) that are retained by the mother cell and not passed on to daughter cells. These forms are not amyloid but require Hsp70 or Hsp104 for their maintenance. (C) The prion-like proteins described by Chakrabortee et al.,^26^ which are induced *de novo* by overexpression of a typically low-abundance protein, although the nature of the resulting altered forms of the protein (yellow) remains to be defined but are not amyloid. Depending on the protein, they either require Hsp104, Hsp90, or Hsp70 to maintain the associated phenotype. In all three cases, the cells in green show the altered phenotype. Reproduced with permission from Tuite M.F. et al.^24^

After discovering and validating PrPSc as a prion, several fungal proteins were identified, in the yeast *Saccharomyces cerevisiae (*Ure2p and Sup35p) and one in the filamentous fungus *Podospora anserina* (HET-s) that showed prion-like properties^89^ i.e., can switch via a self-templating mechanism to one or more alternative amyloid conformations for transmissible prion seeds (Figure 4A). These seeds, sometimes referred to as “propagons”, are then transmitted from mother to daughter cells during mitosis and to haploid spores following meiosis.

Such atypical protein behavior leading to the transgenerational epigenetic inheritance of specific phenotypes in fungi is not restricted to prions. For example, non-transmissible high molecular weight “superassemblies” of the Whi3p protein—referred to as a mnemon^90^—is retained by the mother cell in response to an aborted mating event, giving rise to a form of molecular memory not inherited by daughter cells (Figure 4B). There are also 50 or more low abundance, intrinsically disordered proteins which can generate a range of different, stably inherited phenotypes in yeast when transiently over expressed and share many of the properties ascribed to prions (Figure 4C**)**.^26^

The inheritance of the prion-linked phenotype in fungi requires that the elongating amyloid fibrillar forms of the protein be fragmented prior to cell division by the coordinated action of three chaperones. In the case of prions, three chaperones, Hsp40, Hsp70, and Hsp104 mediate such fragmentation to generate the essential seeds.^91^ A similar process mediated by a functionally related trio of chaperones appears to occur in mammals as well^91^ and Hsp104 has also been implicated in the formation of the other forms of protein-based epigenetic memory but here the role of chaperones is less clear.

Unlike PrPSc, some fungal prions can cf. environment dependent, beneficial traits on their host rather than being detrimental or lethal.^92^ Prions have also been reported in viruses, bacteria, insects, and plants and so might represent a rare but universal mechanism for heritable modulation of protein function and diversity.^93^

What enables a protein to switch to a transmissible prion form can be gleaned in part by interrogation of the primary amino acid sequence.^94^ Examination of various prion-forming proteins suggests that they harbor one or more discrete, low-complexity regions (“domains”) that are essential for the switch to the heritable amyloid form. These prion-forming domains (PrDs) in yeast are enriched in the polar amino acids glutamine and asparagine (i.e., are QN-rich).^95^ However, such a QN-rich region is absent in PrPc and in several other prion-forming proteins, suggesting that QN residues are not essential for prion formation *per se* but likely contribute to the intrinsically disordered structure.^26^ The less prevalent hydrophobic and aromatic amino acids could play important roles in the assembly of prion aggregates.^96^ Remarkably, several hundred proteins in eukaryotes contain sequences showing PrD characteristics and are referred to as “prion-like domains” (PrLDs). Besides possibly defining prion-forming proteins, PrLDs might be important for the formation of a variety of complex, dynamic biomolecular condensates, such as stress granules.^97^

**Figure 5:**
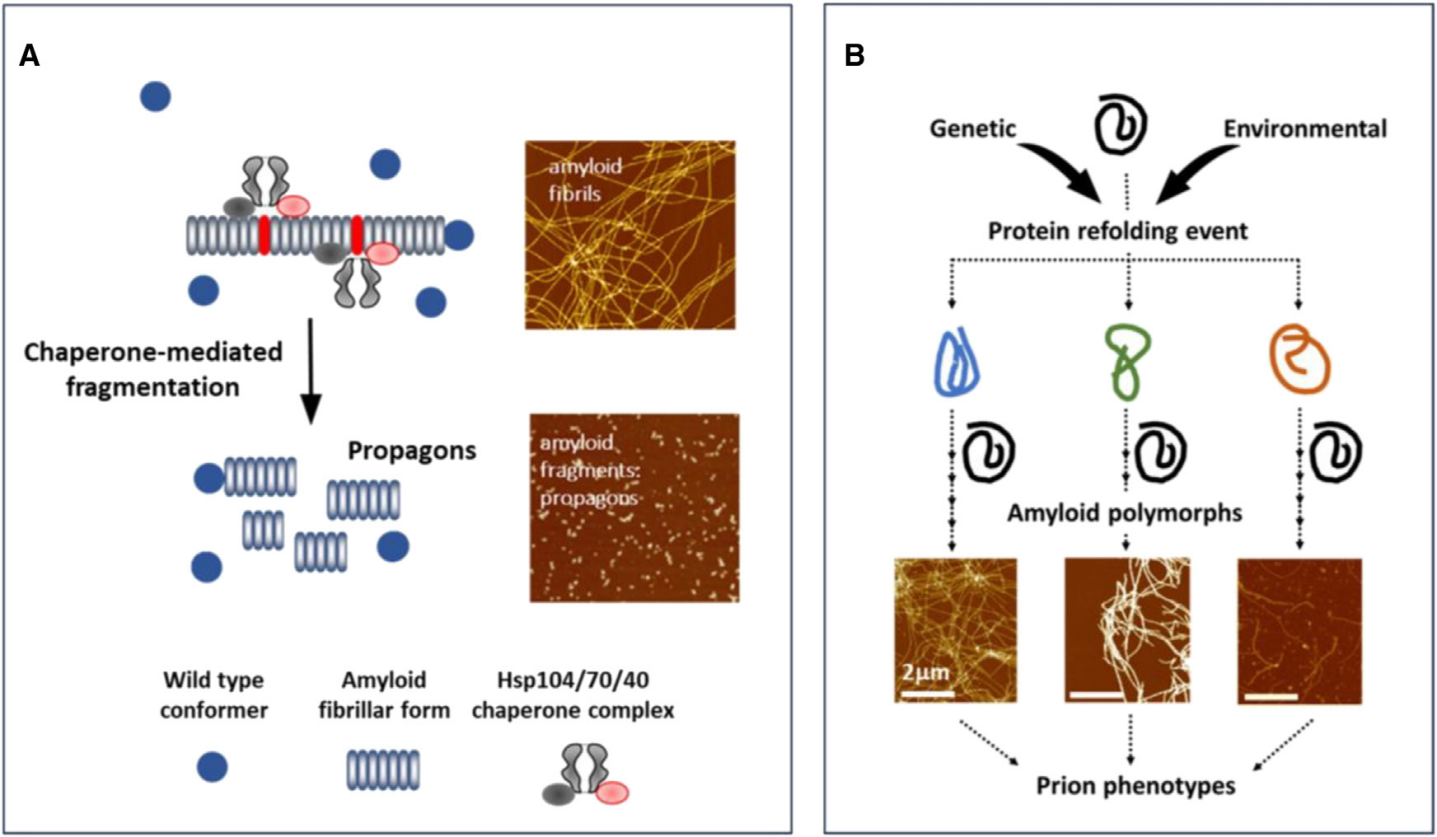
Prions: Proteins mediating transgenerational inheritance (A) Certain proteins can refold to take up an amyloid form, triggered either by changes in the cellular environment or because of a mutational change in the protein’s sequence. As illustrated, the resulting amyloid can take up one of several different structures (amyloid polymorphs) which in turn can give rise to distinct prion-mediated phenotypes. (B) The transmissible form of the prion amyloid is generated by fragmentation of the amyloid fibrillar form of the protein. This fragmentation is mediated by the coordinated action of at least three different molecular chaperones and the resulting transmissible fragments are referred to as prion seeds or propagons. The atomic force microscopic (AFM) images refer to different mutant forms of the yeast Sup35 prion-forming domain (A) or the wild-type form of the same protein (B). AFM images kindly provided by Drs Wei-feng Xue and Ricardo Marchante, University of Kent.

One of the most enigmatic properties of prion-forming proteins is their propensity to refold into an ensemble of alternatively folded transmissible amyloid polymorphs and their respective assembly states^98^ (Figure 5A). Prion polymorphs show both structural and functional diversity and can give rise to different outcomes in terms of pathology for PrPSc in animals (prion strains) or phenotype linked to fungal prions (prion “variants”). This novel type of diversity can occur in response to genetic or environmental triggers and arise without underlying genomic changes. This suggests that there is a common thread with IDP conformational noise although mutations within the prion protein can also lead to different conformational forms of the protein (Figure 5B). Species-specific differences in prion protein sequence may also substantially reduce the potential for inter-species transfer of prions, giving rise to an all-important species barrier.^99^

Prion propagation involves a self-templating mechanism whereby the prion form imposes its structure on the natively folded or partially unfolded form of the same protein. Individual monomers are sequentially added to the ends of the growing fibrils to produce the characteristic fibrillar prion assemblies (Figure 5B). In the case of PrP, this templated conformational switch involves a major rearrangement of the PrPc structure from a largely α helical to a densely packed β-strand-rich configuration, highlighting the connection to fold-switching. However, prion fold switching is driven by multimerization, which is not required for many other fold-switching proteins.

Although the prion-templating mechanism was first proposed over three decades ago, critical details of how prions and their structural variants are formed and propagated are still lacking. There has been considerable debate about the atomic-level structural changes that occur during prion formation, and although a structure for one of the transmissible fungal prions—HET-s of *Podospora anserina*—was solved over a decade ago,^100^ only now are high-resolution atomic structures of *ex vivo* but infectious forms of PrPSc are starting to appear.^101^ These emerging studies support the earlier proposal that PrPSc as well as other amyloids have a distinct, in parallel, in-register intermolecular β sheet (PIRIBS) architecture, with all the structural variants forming the same cross-β interactions, but with differences in, for example, the position of the β-turns.^102^ The availability of high-resolution prion structures, particularly of different prion strains and variants, can be expected to soon shed light on how prions variously impact their host and how they are propagated. For example, do different conformers of a given prion protein interact differently with natural binding partners, whether proteins or nucleic acids? These concepts might also apply to major neurodegenerative diseases such as Parkinson’s and Alzheimer’s disease, where evidence is beginning to emerge of both transmissibility and strain diversity, suggesting that transmission might occur via a prion-like mechanism.^103^ Lastly, it is worth noting that the capacity to form polymorphs is not limited to amyloid-based prions and that, these polymorphs appear to contribute to adaptive diversification of phenotypes in natural settings.^104^

## Impact and implications of the new picture of the protein universe

The discoveries of the last 30 years, in particular, demonstration of the widespread and diverse biological roles of IDPs, amyloid formation, and fold switching, amount to a paradigm shift, revealing major aspects of protein structure and dynamics that had not been fully appreciated previously. These discoveries make it clear that proteins can exist in a continuum of states from ensembles of interconverting conformers to highly stable structures. Proteins are now being looked at from new angles, such as nonlinear dynamics.^19^ Conformational dynamics of ordered/structured proteins has been studied previously, to understand protein folding, catalysis and allostery. However, ordered proteins have not been extensively explored from a dynamical systems perspective that is essential for the study of IDPs given the huge repertoire of conformations sampled by these proteins. Thus, the IDP conformational search process involves a higher-dimensional phase space compared to that of an ordered protein. Folding of ordered proteins is generally considered to occur in relatively simple, “minimally frustrated” or “funnel-like” energy landscapes, but the landscapes of IDPs are far more complex, “highly frustrated”, or “weakly funneled”, with numerous local free energy minima separated by low barriers. Fold-switching proteins can be considered as intermediate between typical ordered proteins and IDPs, with at least two minima reachable with comparable probabilities. Retrospectively, the study of IDPs, fold-switchers, and amyloid states seems to prompt a second look at the landscapes of protein folding in general, suggesting that these landscapes could be more rugged and complex than previously suspected.^105^

The idea that IDPs represent edge-of-chaos systems at the boundary between order and randomness (chaos) with maximum complexity, spurred new theoretical approaches. For example, Waddington’s epigenetic landscape has been invoked as a paradigm in which the landscape represents a high-dimensional state space, and cell fates are determined by stable attractors. This analogy shed new light on how IDP conformational dynamics influences cell fate decisions.^19^

With the advent of powerful artificial intelligence (AI)-based approaches implemented in programs, such as AlphaFold2 and ESMFold, the structures of hundreds of millions of ordered proteins can be predicted with high accuracy.^105^^,^^106^^,^^107^ However, IDPs, amyloids, as well as fold-switching proteins still present the prediction methods with major challenges that need to be addressed.^68^

Until recently, ordered proteins had been the targets of choice for developing effective therapeutics because of their well-defined docking sites.^108^ However, IDPs,^108^ amyloid plaques, or their precursor oligomers^109^ as well as fold-switching proteins^102^ are now increasingly being investigated as potential novel drug targets in several diseases. The discovery that numerous peptides and proteins harbor a latent potential to convert from their native functional states into intractable amyloid aggregates not only further underscores the importance of the protein structure continuum but also provides new insight into mechanisms of several devastating human diseases.^81^ The capability of IDPs/IDRs to form multivalent interactions and undergo LLPS represents a unique molecular mechanism underlying the emergence and biogenesis of numerous biomolecular condensates and MLOs, which are now believed to serve as means of organization and control of intracellular space.^52^

## Conclusions and future directions

The discovery of the widespread and major biological importance of IDPs, fold-switching proteins, amyloids, and prions, calls for new thinking on protein structure and folding. Some IDPs are almost completely disordered, but they are not random coils and exhibit conformational preferences that have major impact on their function.^110^ The study of IDPs and fold-switching proteins and amyloids does not refute Anfinsen’s postulate. Rather, these observations suggest that perhaps, the concept of sequence-determined protein folding should be extended to accommodate these substantial variations exhibited by numerous proteins. It remains true that a protein structure is encoded in its sequence, but at least for fold-switching proteins, IDPs, and amyloid-forming proteins, the same sequence gives rise to different structures depending on environmental conditions. The latest findings increasingly show that this structural malleability is far from being a rare exception but rather is a widespread phenomenon in the protein universe. A common belief that may be considered a corollary of Anfinsen’s postulate is that protein function involves only small conformational shifts, whereas major structural changes represent misfolding that often leads to deleterious protein aggregation. The latest findings on fold-switching proteins and amyloid formation dispel this conviction by showing that both alternative folds of the same protein and aggregates can be functional as well as pathological. The prions that propagate amyloid formation across generations are the ultimate manifestation of these phenomena. Anecdotal evidence of this greater complexity associated with the protein structure/function paradigm was obtained many years ago, and findings of the last few years made it clear that these are not anomalies but major features of the protein universe.

## Acknowledgments

We thank Dr. Ariane Helou, California Institute of Technology, for her thoughtful comments on the manuscript and expert edits. To all our peers whose work we were unable to cite due to space limitations, we extend our sincere apology. Work in the Chiti laboratory was supported by the 10.13039/501100009888Regione Toscana (Bando Ricerca Salute 2018, PRAMA project) and Ministero dell’Università e Ricerca (projects PNRR PE8 Age-IT and PRIN 2020PBS5MJ). The work done by Joseph W. Schafer and Lauren L. Porter was supported by the Intramural Research Program of the 10.13039/100000092National Library of Medicine, National Institutes of Health (LM202011). Work in the Orban laboratory was supported by 10.13039/100000002NIH R01 GM62154 and GM141290 funding, in the Weninger laboratory by a 10.13039/100000002NIH grant GM132263, and that in the Salgia laboratory by a 10.13039/100000002NIH/NCI Cancer Center Support Grant P30CA033572-40 and a 10.13039/100000005DOD grant HT9425-23-1-0581. Shasha Chong is supported by the 10.13039/100000002NIH/NCI under award number P30CA016042, Pew-Stewart Scholar Award, Searle Scholar Award, 10.13039/100010319The Shurl and Kay Curci Foundation Research Grant, Merkin Innovation Seed Grant, The Mallinckrodt Research Grant, The Margaret E. Early Medical Research Trust Grants, and The Alex’s Lemonade Stand Foundation Innovation Grant under award number 1260879.

## Author contributions

P.K. and R.S. conceptualized the manuscript; P.K., V.N.U., L.P., J.W.S, T.-F. C., S.C., F.C., M.T., and E.V.K. and wrote the first draft; J.N.O., K.R.W., J.O., F.C., E.V.K., and R.S. contributed substantially by revising the manuscript; All authors approved the submitted version and are fully accountable for every aspect of the work.

## Declaration of interests

The authors declare no competing interests.
